# CDC WONDER: Trends in Acute Myocardial Infarction Mortality in the United States From 1968 to 2021

**DOI:** 10.1016/j.jacadv.2025.102010

**Published:** 2025-07-17

**Authors:** Saad Ahmed Waqas, Zahra Imran, Dua Ali, Dmitry Abramov, Salim S. Virani, Raheel Ahmed, Abdul Mannan Khan Minhas

**Affiliations:** a Department of Medicine, Dow University of Health Sciences, Karachi, Pakistan; b Division of Cardiovascular Medicine, Loma Linda University Medical Center, Loma Linda, California, USA; c Department of Medicine, Aga Khan University, Karachi, Pakistan; d Department of Population Health, Aga Khan University, Nairobi, Kenya; e National Heart and Lung Institute, Imperial College London, London, United Kingdom; f Section of Cardiology, Department of Medicine, Baylor College of Medicine, Houston, Texas, USA

**Keywords:** acute myocardial infarction, CDC WONDER, mortality trends

**Figure:**
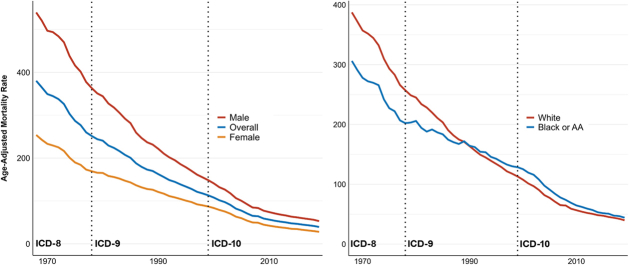

Acute myocardial infarction (AMI) is a prevalent cardiovascular condition with significant public health implications in the United States. Over the last several decades, there have been significant changes in population-based risk factors leading to AMI, which has resulted in a decreased incidence of AMI. Additionally, diagnosis and care of AMI have changed over the last 5 decades, with innovations including widespread adoption of early reperfusion via primary percutaneous coronary intervention, potent antiplatelet therapies, and establishment of care systems significantly reducing AMI mortality.^1^

**What is the clinical question being addressed?**

What are the long-term trends in AMI mortality in the United States from 1968 to 2021?

**What is the main finding?**

AMI mortality rates declined significantly over 5 decades but have slowed in recent years, with persistent disparities by sex, race, and age.

However, prior analyses evaluating AMI trends have often focused on only the last decade or 2 of data, limiting the ability to assess long-term patterns.^2^ A broader evaluation of all available data may offer a more comprehensive perspective on historical trends in AMI mortality, both overall and among key subgroups. Analyzing long-term trends in AMI mortality is essential to understanding the population-level impact of these advancements in management and shifts in risk factor prevalence while also identifying populations where these improvements have been less prominent. This study examines over 50 years of trends in age-adjusted AMI mortality in the United States to provide a comprehensive understanding of mortality trends over time.

Data on deaths in the United States from 1968 to 2021 where AMI was reported as an underlying cause were retrieved using the Centers for Disease Control and Prevention Wide–Ranging Online Data for Epidemiologic Research (CDC WONDER). AMI deaths were identified in individuals ≥25 years of age, with International Classification of Diseases–8th–clinical modification (ICD–8–CM) code 410 for the years 1968 to 1978, ICD–9–CM codes 410 for the years 1979 to 1998, and ICD–10–CM code I21 and I22 from 1999 onward. Age-adjusted mortality rates (AAMRs) and crude rates per 100,000 people with 95% CIs were reported. The 2000 U.S. standard population was used for age standardization.

Mortality trends from 1968 to 2019 were analyzed by the Joinpoint Regression Program (version 5.2.0, developed by the National Cancer Institute). Annual percentage change (APC) with 95% CIs was calculated among intervals identified by the Joinpoint regression. The weighted averages of the APCs were reported as average APCs (AAPCs) and 95% CIs as a summary of the mortality trend across the study period. AAMRs for AMI (only overall) were reported separately for 2020 and 2021 (not included in the Joinpoint regression analysis).

Between 1968 and 2019, there were 11,671,863 deaths due to AMI causes. Overall, the AAMR decreased significantly from 380.3 (379-381.6) in 1968 to 39.4 (39.1-39.6) in 2019 with an AAPC of −4.3% (−4.34% to −4.27%). The AAMR declined from 1968 to 1973 (APC: −3.0% [–3.4% to −2.4%]), from 1973 to 1977 (APC: −5.4% [–6.2% to −4.7%]), from 1977 to 1982 (APC: −2.9% [–3.4% to −1.9%]), and from 1982 to 2001 (APC: −4.0% [–4.1% to −3.9%]). The slowest decline occurred from 1977 to 1982 (APC: –2.9% [–3.4% to −1.9%]), while the steepest decline was from 2001 to 2009 (APC: –7.0% [–7.4% to −6.6%]). In the most recent pre-COVID-19 era, the rate of decline from 2009 to 2019 demonstrated an APC of −3.7% [–4.0% to −3.3%]). In the first 2 years of the COVID-19 pandemic, the AAMR for AMI increased from 39.4 (39.1-39.6) in 2019 to 40.6 (40.4-40.8) in 2020 and further to 41.4 (41.1-41.6) in 2021 (Figure 1A).

Between 1968 and 2019, there were 6,731,226 deaths in males and 4,940,637 deaths in females due to AMI causes. Among males, the AAMRs were consistently higher compared to females, decreasing from 540.0 (537.3-542) in 1968 to 53.0 (52.6-53.4) in 2019. Among females, the AAMR decreased from 253.9 (252.5-255.3) in 1968 to 28.0 (27.8-28.3) in 2019. During the study period, the AAMRs declined among males and females (AAPC: –4.4% [–4.5% to −4.4%] and −4.2% [–4.23% to −4.14%]), respectively; Figure 1A).

Between 1968 and 2019, there were 10,568,623 and 972,022 deaths due to AMI causes in White and Black or African American (AA) populations, respectively. From 1968 to 1988, AAMRs were higher in White individuals compared to Black or AA individuals; however, from 1989 onward, AAMRs were consistently higher in the Black or AA population. Among White individuals, AAMRs decreased from 387.4 (386.1-388.8) in 1968 to 39.8 (39.6-40.1) in 2019. For Black or AA individuals, AAMRs decreased from 306.2 (302.2-310.3) in 1968 to 44.2 (43.4-45.1) in 2019. Overall, the AMI mortality decreased among both White (AAPC: –4.3% [–4.4% to −4.2%]) and Black or AA individuals (AAPC: –3.7% [–3.7% to −3.6%]) during the study period (Figure 1B).

Between 1968 and 2019, there were 248,689 deaths among younger adults (25-44 years), 2,661,599 deaths among middle-aged adults (45-64 years), and 8,761,575 deaths among older adults (65+ years). The decline in AAMRs was observed among older adults, with an AAPC of −4.1% (−4.2% to −4.1%), middle-aged (AAPC: –4.4% [–4.4% to −4.3%]), and younger adults (AAPC: –4.4% [–4.5% to −4.3%]).

This analysis of U.S. national death certificate data demonstrates a significant decline in AAMRs for AMI between 1968 and 2019 across both sexes, Black or AA and White individuals, and all age groups. Throughout the study period, males had higher AAMRs than females, and older adults experienced the highest AAMRs. In later years, AAMRs among Black or AA individuals surpassed those of White individuals.

Our findings are consistent with a recent report using the same database, which evaluated trends in AMI mortality only in the last 2 decades.^2^ By extending the analysis back to 1968, we provide a broader historical perspective, enabling a comparison of contemporary trends with earlier periods. AAMRs for AMI declined consistently throughout the study period, with the decline in AAMR in the 1970s likely attributed to antismoking campaigns and advances in AMI management, such as the widespread adoption of coronary care units.^3^ The sharpest reduction, observed from 2001 to 2009, can be attributed to further advances in medical treatment, including the widespread adoption of primary percutaneous coronary intervention as the preferred reperfusion strategy, which significantly improved AMI survival rates.^1^ However, the decline has slowed in the past decade, raising concern. This slowdown is likely due to an increased prevalence and earlier onset of risk factors like hypertension, obesity, and diabetes mellitus.^4^ Additionally, the high levels of physical inactivity and increasingly sedentary lifestyles in the United States, both of which are well-known contributors to AMI mortality, may also be influencing this trend.

Racial disparities in AMI mortality are also driven by social determinants of health. Initially, AMI mortality was higher in the White population; however, the Black or AA population later surpassed it. Our analysis highlights a slower rate of decline in AMI mortality among Black or AA individuals, suggesting that despite overall advancements in AMI care, the benefits have not been equitably distributed across racial groups, including in the current era. Socioeconomic disadvantages, higher uninsured rates, and unequal access to health care among Black or AA individuals likely contribute to these disparities.^5^ While the exact reasons behind these disparities remain unclear, inequities in prevention, treatment, and access to care likely contribute to the observed trends.

Our analyses have limitations, including the potential for misclassification of the cause of death due to reliance on ICD codes and death certificate data. Additionally, variations in diagnostic criteria, coding practices, and reporting standards over time likely impacted our results.

In conclusion, the decline in AMI mortality rates observed since 1968 has slowed down after 2009. While mortality rates were initially higher in White individuals, they surpassed those in Black or AA individuals in 1989, with persistently elevated rates in males and older adults throughout the study period.

**Figure 1:**
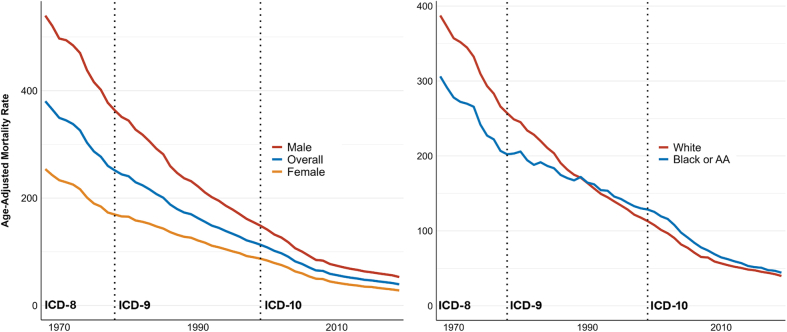
Age-Adjusted Acute Myocardial Infarction Mortality Rates for the Overall Population, Males, and Females, and Black or AA and White Populations Age-adjusted acute myocardial infarction mortality rates for the overall population, males, and females (A), and Black or AA and White populations (B). The dashed vertical lines represent a transition in ICD codes. AA = African American; ICD = International Classification of Diseases.

## Funding support and author disclosures

The authors have reported that they have no relationships relevant to the contents of this paper to disclose.
